# More bang for your byte

**DOI:** 10.1038/sdata.2014.10

**Published:** 2014-05-27

This week, Nature Publishing Group formally launches its newest journal, *Scientific Data*—a peer-reviewed, online-only, open-access publication designed to provide a better way to publish about data.

*Scientific Data* was developed in close collaboration with the scientific community, including researchers who are actively working to promote data standardization and sharing within their own fields, as well as librarians, funders and others interested in wider data sharing. Many have joined our advisory panel or editorial board, and we thank them for their support in bringing *Scientific Data* to fruition. Over the coming months we will be publishing a series of interviews on our blog (http://blogs.nature.com/scientificdata), where they will expand on the challenges in their own communities and how they expect *Scientific Data* to help.

For many of our supporters, the question is no longer whether research data should be shared, but how to make effective data sharing a common and well-rewarded part of research culture. In some fields, well-established data repositories and standards already provide compelling demonstrations of the value of data sharing and reuse. In others data sharing is less standard, although a cultural shift is underway with scientists from all fields coming to expect data to be made public at some point during the research cycle.

There are still, however, substantial barriers to data sharing. One of the biggest is a lack of incentives, especially sharing of well-described, reusable data. A patchwork of funder and journal policies require scientists to share in specific circumstances—alongside their research articles or in association with specific grants. *Nature*-branded journals already have strong, progressive policies here, but there are few rewards for scientists willing to go beyond these requirements—scientists that are committed to sharing a fuller picture of their research data. *Scientific Data* provides a publication venue that credits scientists who share and explain their data. In addition, we understand that quality and credit go hand-and-hand: all our publications are peer-reviewed by expert scientists to ensure that the data described in our journal is well-described, technically sound and of wider use to the scientific community.

At *Scientific Data*, we will judge our success by the degree to which we enrich the scientific literature: by publishing valuable datasets that might not normally get into the open; by setting the standard for quality data publishing; and by increasing the discoverability and reusability of datasets. We believe our first publications set us on a solid path to achieving these goals.

Our launch publications include Data Descriptors—*Scientific Data’s* primary article type—describing previously unpublished datasets and expanding on previous publications. For a compelling example of the former, we encourage you to read the work by Hao *et al.*^1^ describing a timely series of datasets tracking global drought. The authors recently used these data to produce a dramatic visualization of the drought conditions in California in a letter to *Science* ^2^. Using their Data Descriptor, anyone can download the data themselves, generate similar maps for any region of the globe, past or future, and even recalculate the drought metrics using the authors’ own source code.

The publication by Edgar & Stuart-Smith provides an excellent example of a Data Descriptor that builds on previous publications^3^. It is based around the data produced by the Reef Life Survey (http://reeflifesurvey.com/), a unique citizen-science project that uses volunteer divers to survey biodiversity on oceans reefs around the world. These data have already yielded important insights into reef ecology and conservation that have been published in multiple previous articles. In their Data Descriptor, the authors release these data in full and describe the survey procedures and data standardization—essential information for other scientists interested in using these data within their own research. As this project is driven by the interest and good-will of volunteers, it is only natural to release these data back to the public in an open-access publication, and provides a compelling example of the principles that underlie the open science movement.

We invite you to browse our first publications and discover some of the features that maximize the discoverability and reusability of each published dataset, including formal data citations and curated machine-readable metadata in the ISA-Tab format (http://isacommons.org/). You can find out more on our blog.
